# Optimising fast track care for proximal femoral fracture patients using modified early warning score

**DOI:** 10.1308/003588412X13171221501744

**Published:** 2012-05

**Authors:** B Ollivere, K Rollins, R Brankin, M Wood, TJ Brammar, J Wimhurst

**Affiliations:** ^1^Norfolk and Nowich University Hospitals NHS Foundation TrustUR; ^2^Cambridge University Hospitals NHS Foundation TrustUR; ^3^West Suffolk NHS Foundation TrustUR; ^4^Ipswich Hospital NHS TrustUR

**Keywords:** Fast track, Femur, Fracture, Orthogeriatrics

## Abstract

**INTRODUCTION:**

The care for patients with a proximal femoral fracture has been dramatically overhauled with the introduction of ‘fast track’ protocols and the British Orthopaedic Association guidance in 2007. Fast track pathways focus on streamlining patient flow through the emergency department where the guidance addresses standards of care. We prospectively examined the impact these protocols have on patient care and propose an alternative ‘streamed care’ pathway to provide improved medical care within existing resource constraints.

**METHODS:**

Data surrounding the treatment of 156 consecutive patients managed at 4 centres were collated prospectively. Management of patients with a traditional fast track protocol allowed 17% of patients to leave the emergency department with undiagnosed serious medical pathology and 32% with suboptimal fluid resuscitation. A streamed care pathway based on the modified early warning score was developed and employed for 48 further patients as an alternative to the traditional fast track system.

**RESULTS:**

The streamed care pathway improved initial care significantly by treating patients according to their physiological parameters on admission. Targeted medical reviews on admission instead of the following day reduced the rates of undiagnosed medical pathology to 2% *(p=*0.0068) and inadequate fluid resuscitation to 11% (p<0.0001).

**CONCLUSIONS:**

Implementation of a streamed care pathway can allow protocol driven improvement to initial care for patients with a proximal femoral fracture and results in improved access to initial specialist medical care.

Fractured neck of femur occurs in approximately 1 % of all falls in the elderly,^1^ equating to 80,000 fractures per year in the UR.^2^ The all-cause mortality rate following these fractures approaches 50% at one year.^2^ The challenge of providing better care for these patients has been recognised in the NHS through financial incentives with the implementation of ‘best practice’ tariff uplifts, agreed national guidance on best practice (British Orthopaedic Association [BOA] Standards of Trauma), a national audit with published standards and results (National Hip Fracture Database), and the implementation of a variety of ‘fast track’ protocols to ease the burden on emergency departments.

Fast track care for fractured neck of femur patients is a well established system for rapid admission through the accident and emergency (A&E) department in those patients with a presentation of proximal femoral fracture secondary to a mechanical fall.^3^–^5^ Although the fast track pathway var ies between NHS trusts, it is widely accepted that patients should be over 60 years of age,^3^ have a clear history of a mechanical fall and have no underlying serious causative pathology.^6^ Prior to transfer to the ward or direct referral to the orthopaedic receiving team, initial nurse-led management is instigated, which should include analgesia, venepuncture, intravenous fluid administration, diagnostic x-rays and an electrocardiography.^6^

The system was introduced initially to alleviate the waiting time pressures in the A&E department and improve the quality of care for patients. Several previous studies^3^, ^4^, ^7^ have examined the impact of the fast track system on the provision of care and demonstrated significant reduction in waiting times to admission of between 2 hours^3^ and 5.7 hours.^4^ Neither of these studies examined the time from admission to provision of essential care, only the passage of the patient through the emergency department. While fast track systems have addressed patient flow through the A&E department and delays to admission, they have not addressed the quality of care.

There is accumulating evidence that prompt resuscitation^8^ and appropriate medical management decreases complication rates and improves outcomes. The BOA guidelines^9^ have been introduced to improve the standards of inpatient care.

A multidisciplinary fast track approach has been shown to be beneficial to patient outcomes when including input from other medical specialties on admission.^10^ There is, however, some evidence that a rapid admission may not be appropriate for all patient groups and may be predictive of poor outcome.^5^

There have been no recent studies evaluating current standards of care in the emergency department despite the new guidelines. We therefore sought to evaluate and then optimise initial standards of care provided by the fast track protocols while following the BOA guidelines.

## Methods

A prospective, multicentre study was undertaken to evaluate the current initial standards of care for patients attending emergency departments with a diagnosis of fractured neck of femur prior to their admission to the orthogeriatric unit. AH patients attending the emergency department of two large teaching hospitals and two neighbouring district general hospitals were entered prospectively into a three-month study. All four institutions provide care completely in line with current BOA guidance and all four hospitals implement similar protocol driven, nurse-led fast track pathways for patients presenting with a suspected fractured neck of femur.

Data were collected prospectively on a standardised proforma by the admitting orthopaedic doctor at the time of referral to the orthopaedic team. This was supplemented with an additional review of hospital, emergency department and ambulance service notes undertaken the morning following admission. The standard of care was assessed at the time of the referral to the orthopaedic team and therefore only the initial care provided by the existing fast track protocol was being evaluated.

Details of accuracy of diagnosis, adequacy of fluid resuscitation and analgesia administration, recognition and treatment of any serious underlying medical pathology and requests for baseline investigations were used as primary outcome measures. In addition, timings for each intervention were recorded when available. The primary outcome measure of an acceptable standard of care was defined as the patient receiving adequate intravenous fluids and opioid analgesia as well as successful treatment of any serious underlying medical pathology. This was defined as any acute medical pathology that as an isolated diagnosis in its own right would require acute admission under a medical team.

**Figure 1:**
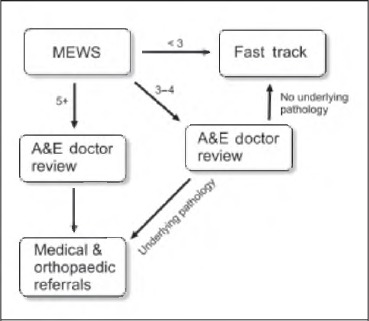
Streamed care pathway based on modified early warning score (MEWS)

Subsequent to analysis of the initial phase of the study, an optimised, revised, ‘streamed care’ pathway was devised and implemented (Fig 1). Streamed care is a fast track management strategy that is determined by the patients’ modified early warning score (MEWS) at admission (Table 1). The study was repeated six months following the implementation of this pathway to examine its effect. Identical outcome measures were used.

**Table 1:** Standards of medical care provided by fast track and streamed care groups

	Fast track care	Streamed care	p-value
Patients	156	48	
Missed underlying medical pathology	27 (17%)	1 (2%)	**0.0068**
Analgesia administered	102 (54%) (n=102)	39 (82%)	**0.048**
Fluid resuscitation started	56 (32%)	43 (89%)	**< 0.0001**

**Table 1:** Modified early warning score: The scores for each parameter are summed.

Parameters	Score						
	3	2	1	0	1	2	3
Respiratory rate (breaths per minute)		<8		9–14	15–20	21–29	>30
Pulse (beats per minute)		<40	41–50	51–100	101–110	111–129	>130
Systolic blood pressure (mmHg)	<70	71–80	81–100	101–199		>200	
Urine output (ml/kg/hr)	nil	<0.5					
Temperature (°C)		<35.0		35.1–37.5	37.6–38.5	>38.6	
AVPU scale				Alert	Voice	Pain	Unresponsive

The streamed care pathway makes use of the validated MEWS system to establish the patients’ physiological condition. Patients with a normal score (<2) are nurse managed according to a modified fast track protocol while patients with an intermediate score (3–5) are assessed by an A&E doctor and then either managed according to the protocol or referred on to medical and orthopaedic teams as appropriate. Patients with a higher score are managed by the A&E doctor acutely, and a medical and orthopaedic referral is mandatory.

Statistical analysis was undertaken using Prism® v5 (GraphPad Software, La Jolla, CA, US). Statistical significance was determined using a two-tailed Fisher’s exact test to compare the event rate for categorical data. Significance levels are given to two significant figures. Outcome data are given as a percentage with 95% confidence intervals. A p-value of <0.05 was defined as significant in all cases.

## Results

Over a three-month period, 156 patients (79 from the teaching centres and 77 from the district generals) were recruited. No patients were lost to follow-up although a single patient died within the 24-hour period of the study. Hospital records were available to review for all patients in the study. Following revision of the fast track protocol to a streamed care pathway, the study was repeated and 48 patients were recruited in a single centre in a new three-month study period.

### Fast track protocol

All patients had received appropriate medical management following review by the receiving orthopaedic team. However, not all patients received all appropriate interventions, nor were definitive medical diagnoses reached while in the emergency department. Opioid analgesia was administered in the emergency department or in the ambulance in 54% of fast track patients (102/156), intravenous fluid resuscitation was administered in the emergency department in 56% of patients (56/156). A serious medical diagnosis remained undiagnosed in 17% of patients (27/156) by the time they were referred to the orthopaedic team. Of those missed diagnoses, H patients had had a myocardial infarction or an acute coronary syndrome, 8 had decompensated congestive cardiac failure or hypoxia, 4 had a collapse and loss of consciousness, 1 had overwhelming sepsis and 1 was coagulopathic.

### Streamed care pathway

Forty-eight patients were managed according to the streamed care pathway during the second half of the study in a single trust. The introduction of the streamed care pathway resulted in statistically significant improvement in standards of care during the patients’ stay in the emergency department. The rate of missed significant medical pathology fell to 2% (p=0.0068) and there were significantly higher rates of fluid administration (89%, pcO.OOl) and analgesia (p=0.048) than in the original fast track group. The streamed care protocol resulted in 25% of patients («=11/48) receiving a medical review in the emergency department. Of these H, 10 received treatment subsequently from the reviewing clinician that would otherwise likely have been delayed until the orthogeriatric review.

There were no significant differences in rates of venepuncture (p>0.05), electrocardiography recording (p>0.05) or breaches of the four-hour wait (p>0.05) between the two groups.

The MEWS system as implemented in the streamed care pathway in this series was 91% sensitive and 91% specific for detection of significant underlying medical pathology. There were no significant differences in any measured aspect of care between the streamed care and original fast track groups.

## Discussion

When interpreting these results, it is important to remember that they relate only to the provision of care within the first four hours of stay and that there is little evidence surrounding appropriate timings for medical interventions. While this study demonstrates a particularly poor standard of care during the patient’s stay in the emergency department, it seems likely that this is representative of standards of care across the country as these data were collected from four different trusts and the standards of care were similar in each trust. AH of our patients received all the required care following review by the orthopaedic team.

The current fast track pathway does not meet the needs of nearly 20% of patients, who consequentially suffer a needless delay to the start of treatment for their medical pathology. Use of the MEWS system to allocate patients to different care streams improved initial management significantly in all examined outcome measures.

Although much debate surrounds factors that influence outcomes in management of patients with a proximal femoral fracture, delays in administration of adequate analgesia are far from ideal. While there is controversy about the most effective analgesia,^11^ it is clear that strong, long acting analgesia should be the gold standard although not all patients will require opioid analgesia during their stay in the emergency department.

The poor rate of analgesia administration (57%) in the original fast track study group gave us cause for concern. The significant rise in analgesia administration (82%, p=0.048) seen with initiation of the streamed care pathway suggests that at least some patients managed with the fast track protocol would have benefited from further analgesia administration while they were in the emergency department. This improvement is likely to be due to the design of the MEWS system. Patients who are in pain will be tachycardie and tachypneic, which in this protocol will trigger a medical review in our pathway.

Blood loss from a femoral neck fracture may be in excess of U^12^ in addition to any dehydration associated with delay in raising the alarm. Early administration of intravenous fluids and maintenance of good hydration has been shown to improve outcome and reduce complications^8^ in patients with a fractured neck of femur. The nature of the patient population with high rates of congestive cardiac failure is such that a simple protocol driven approach is likely to replace fluids inadequately. The jump in fluid administration seen with the streamed care protocol probably represents appropriate aggressive fluid resuscitation following selected medical review in hypovolaemic patients.

The number of patients presenting with serious underlying medical pathology in addition to a fractured neck of femur is difficult to estimate. Nevertheless, approximately 57% have underlying cardiovascular co-morbidities and 16.5% chronic respiratory disease.^13^ While an alarming number of patients in this series left the A&E department with unrecognised life threatening pathology, all of these patients received appropriate treatment when seen by the receiving team. It is possible that the delay in recognition of pathology would not have a significant impact on eventual outcomes. Although there is no specific evidence relating to delay in treatment of underlying medical pathology in patients with a neck of femur fracture, there is evidence that co-morbidities predict outcomes^14^ and that rapid treatment of coronary syndromes, pneumonia and septicaemia is likely to improve outcomes.

The small sample size and short follow-up duration as well as the diagnoses being too varied made it not possible to formally analyse the outcome by underlying pathology.

This is a significant limitation to the study.

The streamed care pathway relies on the local trust’s MEWS system to select an appropriate care pathway for the patient. Early warning scores are based on the patients’ physiological observations (Table 1) and have been shown to be a reliable measure of a patient’s overall physiological status^15^ and to be predictive of need for admission and subsequent outcome.^16^ The streamed care pathway uses the MEWS system to manage patients according to their physiological status. Patients with a low score (<2) are managed with a traditional nurse-led fast track pathway. Patients presenting with an intermediate score (5–4) are assessed by the A&E doctor and then managed appropriately. A high score (>5) triggers an urgent A&E doctor review as well as a simultaneous referral to the medical and orthopaedic receiving teams.

Implementation of streamed care had a significant effect on the studied outcomes. There were significantly fewer incidences of missed underlying medical pathology; significantly more patients were handed over to the receiving team, and received strong analgesia and fluids in the emergency department. While striking, these improvements could also be due to the ‘audit effect’, where observation of practice encourages adherence to protocols. For this reason, the second part of the study was conducted six months after the new protocol was implemented. In light of the results of this pilot of a streamed care pathway, all three of the other centres are in the process of implementing a similar system.

It is possible that the standards of care provided by the fast track protocols examined in this study are not reflective of the wider picture across the NHS. However, the inclusion of four centres around one large region, using established fast track protocols and prospective data collection, makes this less likely. It is important, however, to appreciate that the net effect of this is a delay in treatment that could have been started earlier in the emergency department, not a complete lack of treatment altogether.

Our results contrast strongly with previous studies on the effects of fast track care. This may be in part due to our evaluation of care provided as a primary outcome measure rather than time spent in the emergency department. Differences observed between the current and previous studies could also be explained by the changes in medical staffing and provision of emergency care necessitated by the Hospital at Night system and the European Working Time Regulations.

This study is limited in that we are unable to state conclusively that better care provided by the streamed care pathway had a positive impact on eventual patient outcomes as the study was designed to look at care in the first 24 hours of admission. A further study with greater numbers and a longer follow-up duration would be required to demonstrate this.

## Conclusions

Fast track protocols may compromise early medical management of patients with a fractured neck of femur and re suit in substandard care. We recommend that all patients should be treated based on their individual physiological status. The standard and timing of medical care provided by streaming care according to the MEWS system is significantly higher than that provided by a traditional fast track system. Adoption of this or a similar pathway across the NHS would improve standards of care for this patient group without using additional healthcare resource.

## References

[1] (1996). Etiology and prevention of agerelated hip fractures. Bone.

[2] (2002). Mortality after admission to hospital with fractured neck of femur: database study. BMJ.

[3] (1996). ‘Fast tracking’ patients with *a* proximal femoral fracture. J Accid Emerg Med.

[4] (2003). Reduction of waiting times in A&E following introduction of ‘fast-track’ scheme for elderly patients with hip fractures. Injury.

[5] Clague JE Craddock E Andrew G et al. Predictors of outcome following hip fracture. Admission time predicts length of stay and in-hospital mortality Injury2002; 33 1 6 11879824 10.1016/s0020-1383(01)00142-5

[6] (2003). Reduced delays in A&E for elderly patients with hip fractures. Ann R Coll Surg Engl.

[7] (2000). Audit of the effect of *a* fast tracking protocol on transfer time from A&E to ward for patients with hip fractures. Injury.

[8] (1997). Intraoperative intravascular volume optimisation and length of hospital stay after repair of proximal femoral fracture: randomised controlled trial. BMJ.

[9] British Orthopaedic Association BOAST 1: Hip Fracture In the Older Person London BOA 2007

[10] (2008). A comprehensive hip fracture program reduces complication rates and mortality. J Am GerlatrSoc.

[11] (2008). A national survey into the peri-operative anaesthetic management of patients presenting for surgical correction of a fractured neck of femur. Anaesthesia.

[12] (1990). ABC of major trauma. Management of limb injuries. BMJ.

[13] (2009). Hip fractures after falls in hospital: *a*retrospective observational cohort study. Injury.

[14] (2008). Development and validation of a preoperative scoring system to predict 30 day mortality in patients undergoing hip fracture surgery. BrJAnaesth.

[15] (2006). The value of Modified Early Warning Score (MEWS) in surgical in-patients: a prospective observational study. Ann R Coll Surg Engl.

[16] (2008). Modified early warning score predicts the need for hospital admission and inhospital mortality. Emerg Med J.

